# Exploration of changes in spatial chondrocyte organisation in human osteoarthritic cartilage by means of 3D imaging

**DOI:** 10.1038/s41598-021-89582-w

**Published:** 2021-05-07

**Authors:** Marina Danalache, Kevin Ralf Beutler, Bernd Rolauffs, Julius Michael Wolfgart, Florian Christof Bonnaire, Stefan Fischer, Imke Greving, Ulf Krister Hofmann

**Affiliations:** 1 Department of Orthopaedic Surgery, University Hospital of Tübingen, Hoppe-Seyler-Strasse 3, 72076 Tübingen, Germany; 2 Medical Faculty of the University of Tübingen, 72076 Tübingen, Germany; 3 G.E.R.N. Tissue Replacement, Regeneration and Neogenesis, Department of Orthopedics and Trauma Surgery, Medical Center - Albert-Ludwigs-University of Freiburg, Faculty of Medicine, Albert-Ludwigs-University of Freiburg, 79108 Freiburg, Germany; 4 Department of Evolutionary Biology of Invertebrates, University of Tübingen, 72076 Tübingen, Germany; 5 Institute of Materials Research, Helmholtz-Zentrum Geesthacht, Geesthacht, Germany; 6 Present Address: Department of Orthopaedic Surgery and Traumatology, Spital Thurgau AG, Spitalcampus 1, 8596 Münsterlingen, Switzerland; 7 Present Address: Tübingen Structural Microscopy (TSM), Center for Applied Geoscience (ZAG), University of Tübingen, 72076 Tübingen, Germany

**Keywords:** Cell migration, Cellular imaging, Cells, Chondrocytes, Osteoarthritis

## Abstract

Using two-dimensional top-down view microscopy, researchers have recently described chondrocytes as being spatially arranged in distinct patterns such as strings, double strings, and small and large clusters. Because of the seeming association of these changes with tissue degeneration, they have been proposed as an image-based biomarker for early osteoarthritis (OA) staging. The aim of our study was to investigate the spatial arrangement of chondrocytes in human articular cartilage in a 3D fashion and to evaluate the 3D changes of these patterns in the context of local tissue destruction. Decalcified femoral condyle resections from the load-bearing area were analysed in 3D for their spatial chondrocyte organisation by means of fluorescence microscopy and synchrotron-radiation micro-computed tomography (SR-µCT). In intact cartilage chondrocyte strings can be found in the superficial, transitional and deep zones. The proposed pattern changes accompanying tissue destruction could be located not just along the surface but also through all layers of cartilage. Each spatial pattern was characterised by a different cellular density (the only exception being between single and double strings with *p* = 0.062), with cellular density significantly increasing alongside the increase in local tissue degeneration as defined by the chondrocyte patterns. We can thus corroborate that the proposed cellular spatial changes are a three-dimensional function of local tissue degeneration, underlining their relevance as an image-based biomarker for the early diagnosis and description of OA.

Clinical trial registration number: Project number of the ethics committee of the University of Tübingen:171/2014BO2.

## Introduction

Osteoarthritis (OA) as degenerative joint disease is one of the leading causes of pain and disability in adults worldwide. It is characterised by an irreversible complete destruction of the articular cartilage. On a microscopic level, articular cartilage is composed of sparsely scattered chondrocytes embedded in an expansive extracellular matrix (ECM). This matrix is morphologically subdivided into the superficial, transitional and deep zone and, at the interface to the subchondral bone, the calcified tide mark^1^. With OA initiation and progression the tissue surface becomes macroscopically roughened as fissuring and fibrillation of its collagen matrix take place^2–4^.

Recent studies have indicated that at its surface, articular cartilage contains characteristic spatial chondrocyte patterns that are specific for each layer and joint^5–7^. The exact orientation of the patterns seems to depend on the joint-specific mechanical loading mechanism^6,8^. How this spatial organisation is established is still unknown^9^. In the femoral condyle, with initiation and progression of OA, the chondrocytes' spatial organisation at the cartilage surface changes from strings to double strings^10^, then to small or even large clusters^7^ (Supplementary Figure 1). S trings are defined as chondrocytes aligned in a line, double strings as two parallel strings and clusters as a circular arrangement of chondrocytes^6^. Due to a seeming association of superficial chondrocyte arrangement and OA changes of the cartilage, it had been suggested that chondrocyte arrangement may act as an image-based biomarker for OA-triggered events and thus be used to diagnose early OA^11^. Recent studies could even demonstrate the functional relevance of these spatial patterns establishing a strong correlation between local tissue elasticity and the locally predominant spatial pattern^12,13^. Analysing the cartilage surface in a top-down view only might, however, lead to perceived changes of cellular organisation already as a result of deeper tissue layers made visible to top-down observation by superficial erosion. An ascending string following the collagen arcades might, for example, be perceived as a single cell or a pair of cells. A full understanding of the changes in spatial chondrocyte arrangement in OA cartilage therefore requires a three-dimensional (3D) approach that examines the tissue throughout all tissue layers. Using healthy bovine cartilage, Zehbe et al. had already demonstrated the feasibility of identifying single chondrocytes using synchrotron radiation micro-computed tomography (SR-µCT)^14,15^.

**Figure 1:**
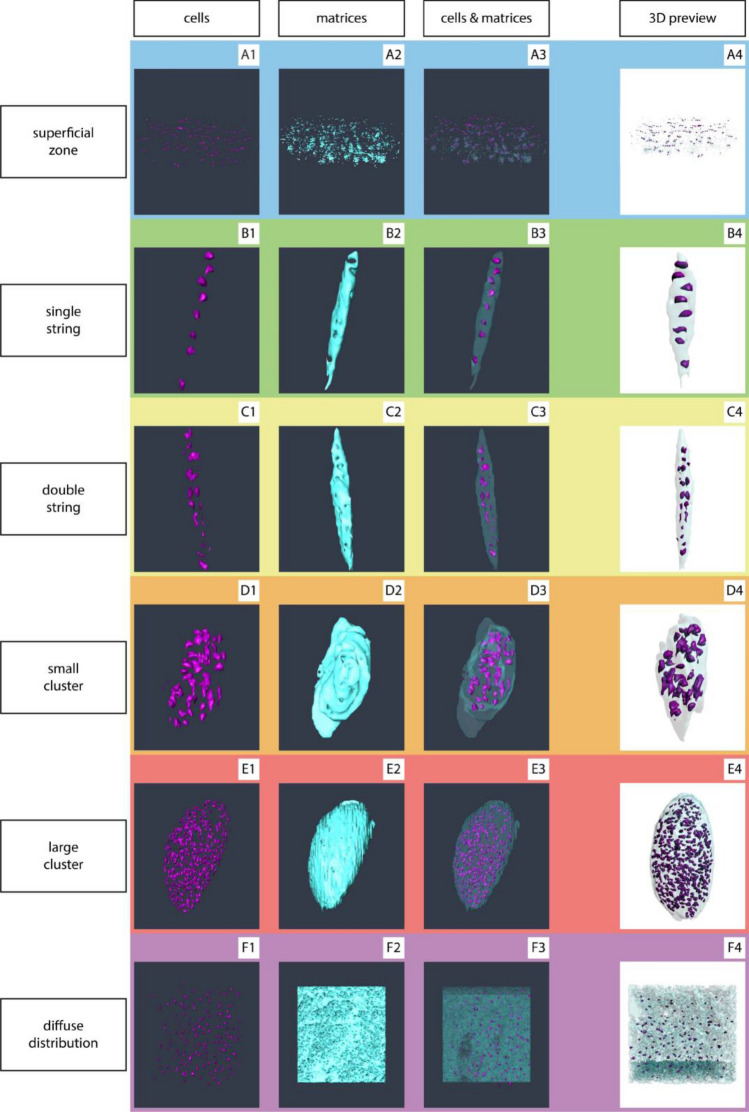
Spatial chondrocyte patterns imaged by µCT. Nuclei are depicted in purple (1), the extranuclear space within the lacuna/chondron is shown in light blue (2). (3) Showing the merged spatial patterns, and (4) the interactive 3D reconstructions of the different spatial patterns. In case of a diffuse cellular distribution, no lacuna/chondron is present any longer but instead the extracellular matrix is depicted. This figure was created using Amira 6.0 (https://www.thermofisher.com/ch/en/home/industrial/electron-microscopy/electron-microscopy-instruments-workflow-solutions/3d-visualization-analysis-software/amira-life-sciences-biomedical.html), MeVisLab (https://www.mevislab.de/mevislab) and Adobe Illustrator CS6 (https://www.adobe.com/ch_de/products/illustrator.html).

The aim of our study was to investigate the spatial arrangement of chondrocytes in human articular cartilage in a 3D fashion and to evaluate the 3D changes of these patterns in the context of local tissue destruction. Since cellular organisation had been shown and validated in the topmost layer of articular cartilage as a 2D phenomenon, we hypothesised that it is in fact a 3D feature of cartilage destruction during OA pathogenesis (Fig. 1).

## Results

### Histologic 2D analysis of spatial chondrocyte patterns

**Figure 2:**
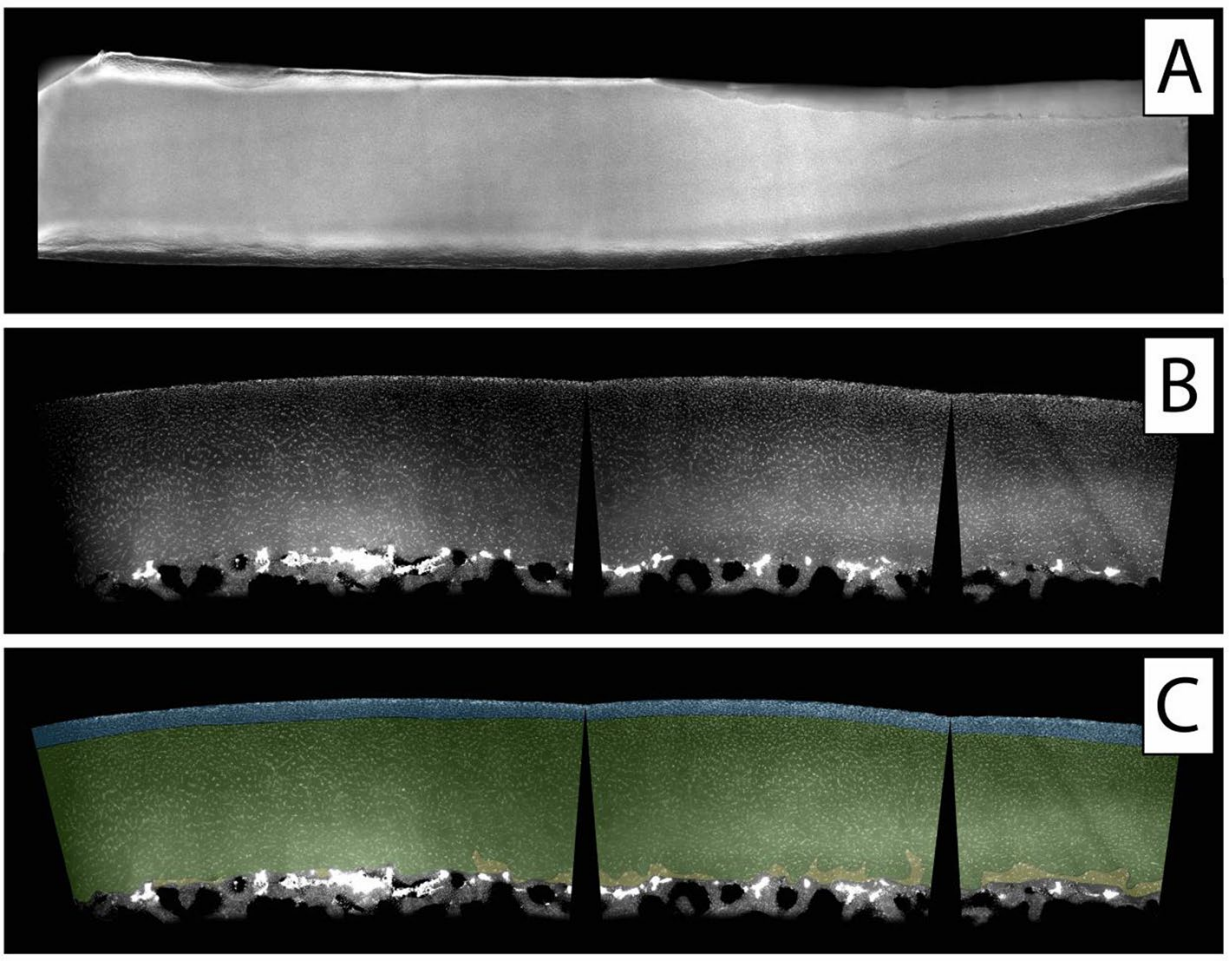
Top-down (**A**) and side view (**B**,**C**) of a healthy cartilage sample. (**C**) Zonal colour labelling of the predominant spatial chondrocyte arrangement: blue—superficial zone, green—single strings, yellow—double strings, grey—subchondral bone. The sample still shows an intact superficial zone. Underlying is a thick layer of healthy cartilage composed of single strings. Only at the transition to the subchondral bone some isolated double strings can be detected. The black wedges are a digital artefact of straightening the image. This figure was created using Adobe Photoshop CS6 (https://www.adobe.com/ch_de/products/photoshop.html) and Adobe Illustrator CS6 (https://www.adobe.com/ch_de/products/illustrator.html).

**Figure 3:**
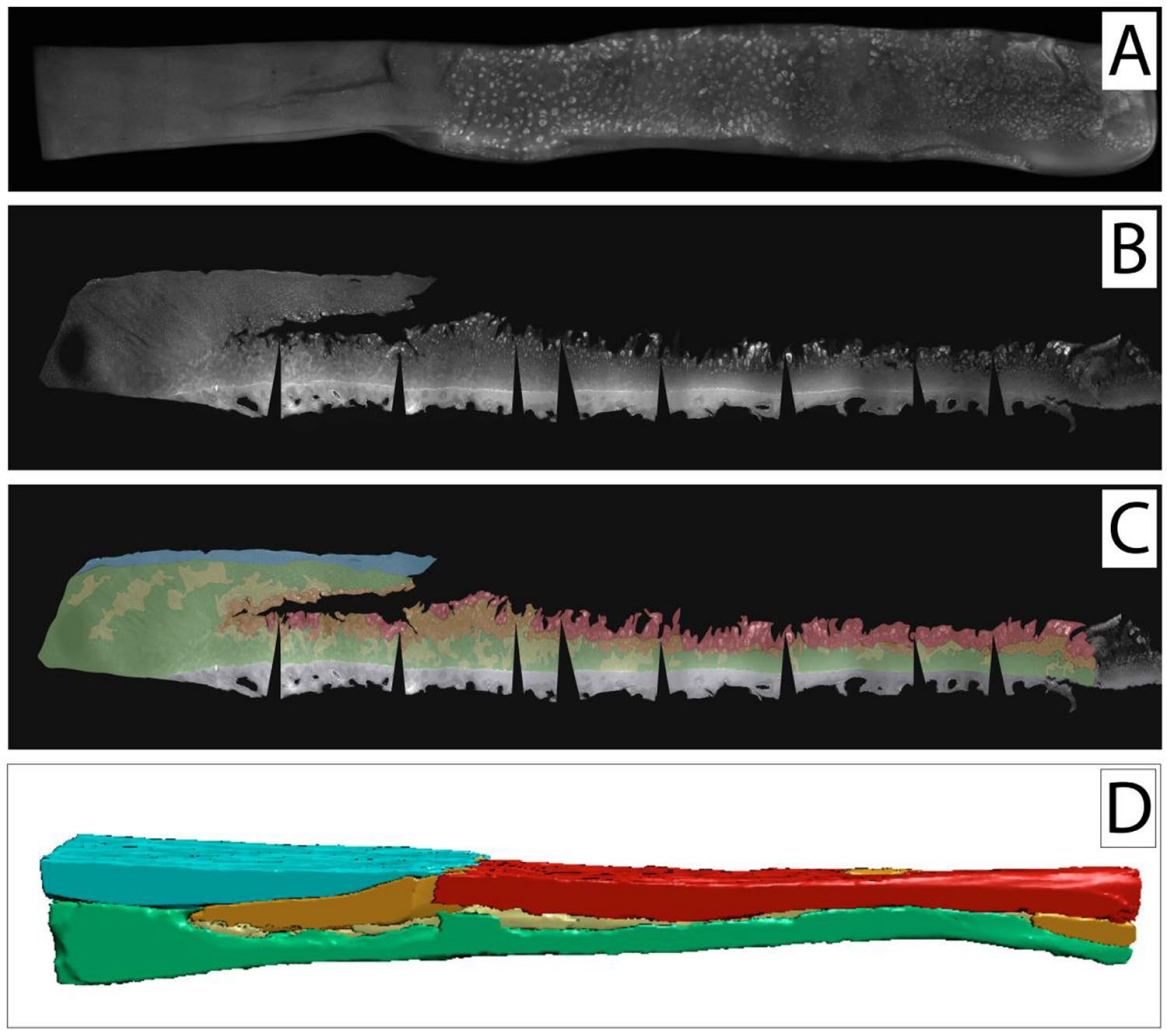
Top-down (**A**) and side view (**B**,**C**) of cartilage specimen I and the corresponding SR-µCT image rendering (**D**). (**C**,**D**) Zonal colour labelling of the predominant spatial chondrocyte arrangement: blue—superficial zone, green—single strings, yellow—double strings, orange—small clusters, red—large clusters, purple—diffuse, grey—subchondral bone. On the left side, in macroscopically intact cartilage, an intact superficial zone with underlying single strings with few areas of double strings can be seen. On the right side, the eroded remaining cartilage shows large clusters in the defect area. Small clusters surrounded by double strings are embedded in the fissure that cuts deep at the transition between these two extrema. The µCT-image confirms these findings throughout all tissue layers also in the full width of the analysed sample. The black wedges are a digital artefact of straightening the image. This figure was created using MeVisLab (https://www.mevislab.de/mevislab), Adobe Photoshop CS6 (https://www.adobe.com/ch_de/products/photoshop.html) and Adobe Illustrator CS6 (https://www.adobe.com/ch_de/products/illustrator.html).

**Figure 4:**
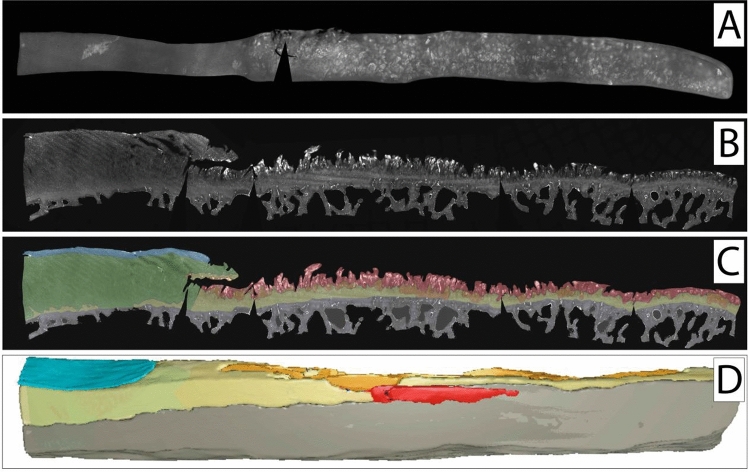
Top-down (**A**) and side view (**B**,**C**) of cartilage specimen II and the corresponding SR-µCT image rendering (**D**). (**C**,**D**) Zonal colour labelling of the predominant spatial chondrocyte arrangement: blue—superficial zone, green—single strings, yellow—double strings, orange—small clusters, red—large clusters, purple—diffuse, grey—subchondral bone. On the left side, in macroscopically intact cartilage, single strings are predominant in the deep zone underlying an intact superficial zone. On the right side, the eroded remaining cartilage shows large clusters in the defect area, with smaller clusters at the transition to the underlying double strings in the tide mark. Due to the thinned-out cartilage layer some subchondral bone had to be left in place to ensure the stability of the specimen. The black wedges are a digital artefact of straightening the image. This figure was created using MeVisLab (https://www.mevislab.de/mevislab), Adobe Photoshop CS6 (https://www.adobe.com/ch_de/products/photoshop.html) and Adobe Illustrator CS6 (https://www.adobe.com/ch_de/products/illustrator.html).

**Figure 5:**
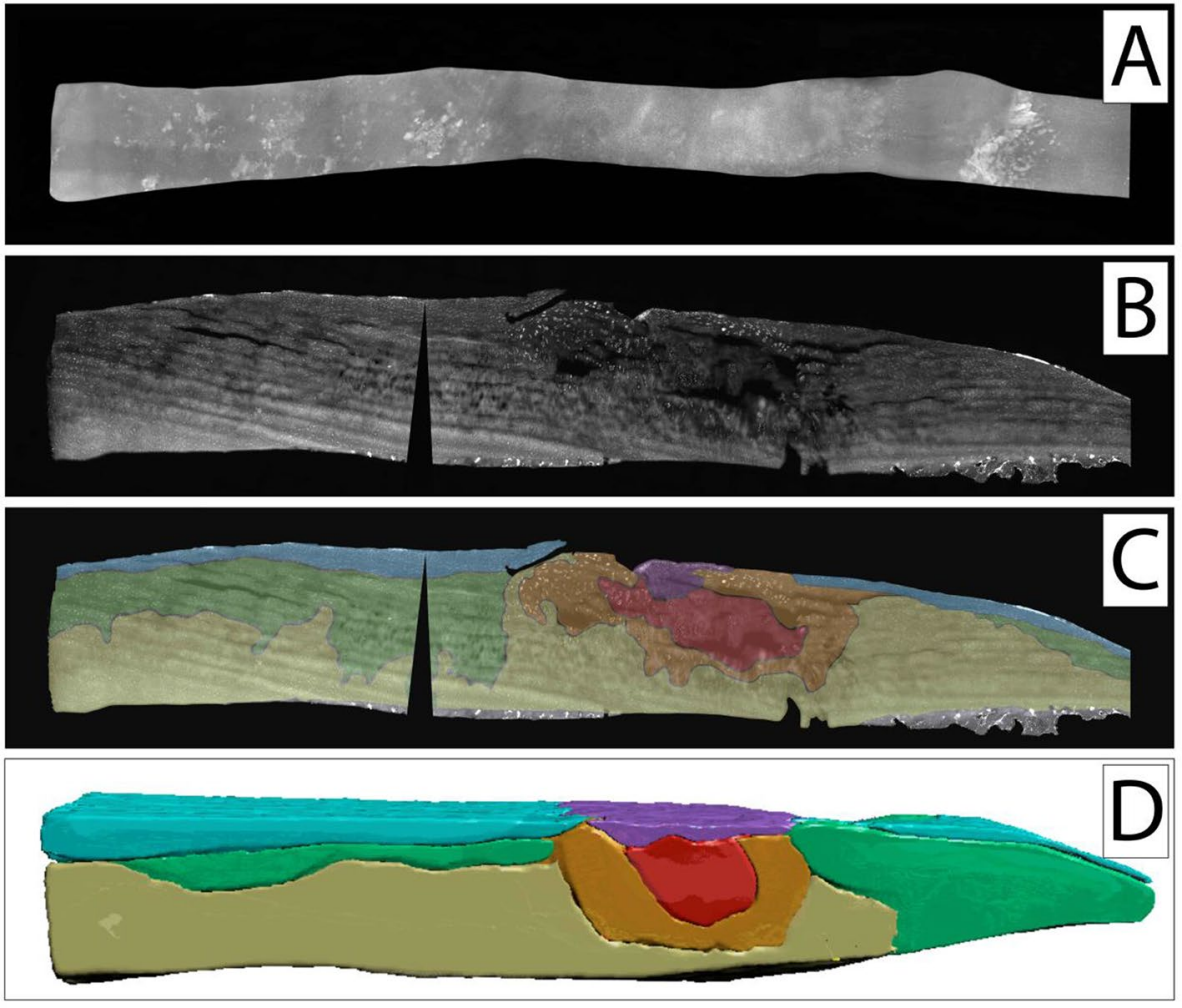
Top-down (**A**) and side view (**B**,**C**) of cartilage specimen III and the corresponding µCT image rendering (**D**). (**C**,**D**) Zonal colour labelling of the predominant spatial chondrocyte arrangement: blue—superficial zone, green—single strings, yellow—double strings, orange—small clusters, red—large clusters, purple—diffuse, grey—subchondral bone. The intact cartilage shows the superficial zone, with single strings in the underlying area in the superficially deep zone and double strings deeper down. In the centre is a macroscopic cartilage lesion from the superficial zone to the transitional zone, also partly including the deep zone. The centre of the lesion is composed of large clusters surrounded by small clusters. Around this area, double strings can be found. Single strings are farther away in macroscopically intact cartilage. Interestingly, the superficial centre of the defect is covered with a scar-like plug of densely packed and highly disorganised cells. In the µCT 3D segmentation it can be clearly recognised how the different patterns arrange themselves in their corresponding order in a concentric fashion. The black wedge is a digital artefact of straightening the image. This figure was created using MeVisLab (https://www.mevislab.de/mevislab), Adobe Photoshop CS6 (https://www.adobe.com/ch_de/products/photoshop.html) and Adobe Illustrator CS6 (https://www.adobe.com/ch_de/products/illustrator.html).

To illustrate spatial chondrocyte organisation in cartilage not affected by OA or trauma, one healthy cartilage sample was analysed in the top-down view (Fig. 2A) and the side-view (Fig. 2B,C) showing all cartilage layers intact with mostly single strings following the arciform architecture of the cartilage. When analysing the samples obtained from patients with OA, all predescribed cellular patterns were visible already in all of the histologic top-down views at the surface of the specimens (Figs. 3A, 4A, 5A). Looking at these samples in the side-view, the arciform architecture could clearly be identified also in the osteoarthritic cartilage samples in areas with macroscopically intact cartilage (Figs. 3B, 4B, 5B). Of note, all described cell patterns could not just be identified in the surface area but in all cartilage layers thus being not layer specific (Figs. 3B, 4B, 5B).

### SR-µCT: 3D analysis of spatial chondrocyte patterns

The SR-µCT imaging of the osteoarthritic cartilage samples allowed to clearly identify individual chondrocytes. Performing the segmentation and rendering techniques described above, all histologically predescribed chondrocyte spatial patterns could be reidentified, isolated, and interactive 3D-models could be generated (Fig. 1, Supplementary Figure 2&3) thus demonstrating the aptitude of the technique for the study question.

When analysing the side-view sections with the colour-coding of the different patterns (Figs. 3, 4, 5C) and the corresponding colour-coded SR-µCT renderings (Figs. 3, 4, 5D) a clear picture presented itself: When looking at macroscopically highly disrupted cartilage, the predominant patterns were small and large clusters. When the cartilage was eroded well down into the deep zone, it was in the remaining fissured tissue that these patterns could be observed (Figs. 3, 4). Large clusters could thereby contain up to several hundreds of chondrocytes. Interestingly, in one cartilage sample with a major tissue lesion in the transitional and deep zones, the organisational changes could be found surrounding the lesion not just along the surface in the horizontal plane, but also through all cartilage layers in the vertical plane (Fig. 5). With a diffuse chondrocyte tissue lump as a plug or a scar in the defect zone, large clusters were found at the heart of the damaged tissue, surrounded by smaller clusters. Encompassing this zone, double strings were the dominant cellular pattern, and strings were found only farther away from the lesion in macroscopically intact cartilage. The cellular changes could thus be observed as 3D and not just 2D tissue changes with respect to local cartilage damage.

### Cellular density—a distinctive feature of chondrocyte spatial patterns and cartilage degeneration

**Figure 6:**
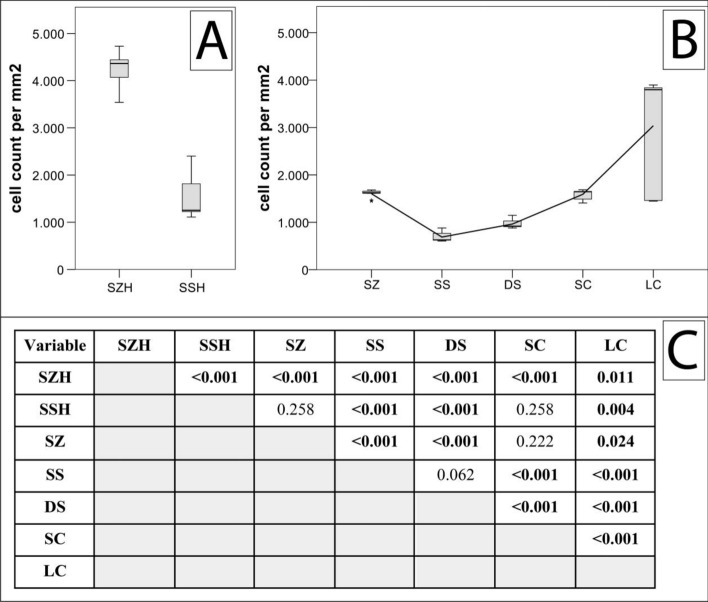
Cellular density in healthy (**A**) and osteoarthritic (**B**) cartilage as a function of cellular organisation. *P*-values are listed in (**C**) with significant *p*-values denoted in bold. Significance was determined on an FDR-adjusted alpha (for absolute cell count values, see Table 1; for exact FDR-adjusted alphas, see supplementary Table 1). Abbreviations: SZH—superficial zone healthy cartilage; SSH—single strings healthy cartilage; SZ—superfical zone; SS—single strings; DS—double strings; SC—small clusters; LC—large clusters. Significant differences are listed in the table below. For zonal labelling according to the predominant regional pattern, see also supplementary Figure 5. This figure was created using SPSS Statistics 23 (https://www.ibm.com/ch-de/products/spss-statistics), Microsoft Office Word (https://www.microsoft.com/de-ch/microsoft-365/word) and Adobe Illustrator CS6 (https://www.adobe.com/ch_de/products/illustrator.html).

When counting the cells in representative areas for the different patterns (Supplementary Figure 5), each specific zone was characterised by a distinct cellular density (Fig. 6, Table 1): the highest physiological values were found in the superficial zone in both the healthy and the osteoarthritic samples, and the lowest values were seen in cartilage areas other than the superficial zone in regions presenting with single strings. With increasing pathological spatial cellular organisation, cellular density also increased from double strings to small clusters and finally to large clusters. Considering the different values obtained from the different spatial patterns of osteoarthritic specimens in an ordinal sense, all differences were statistically significant on the basis of an FDR-adjusted alpha, the exception being between single and double strings (*p* = 0.062).

**Table 1:** Cellular density as a function of cellular organisation.

Cellular organisational pattern	Median (interquartile range) (cells/mm^2^)
Superficial zone healthy cartilage	4363 (590)
Single strings healthy cartilage	1252 (823)
Superficial zone	1622 (126)
Single strings	628 (157)
Double strings	918 (124)
Small clusters	1646 (188)
Large clusters	3798 (2393)

## Discussion

As chondrocytes are key players for normal articular cartilage homeostasis, functional cell loss or change of their normal organisation and distribution could be a primary trigger that leads to cartilage failure in OA. In the present study, we investigated chondrocyte spatial organisation within the articular cartilage from human femoral condyles, with a special focus on the 3D distribution of the predescribed cellular patterns across all cartilage layers. We observed these pattern changes not just in the horizontal topmost layer but also in the vertical plane as a 3D-function around cartilage lesions.

As previously described in 2D^6,10^ and corroborated now in a 3D approach, we found strings of cells lying horizontally in the intact superficial zone and ascending vertically along the arcades in the deep zone in microscopically intact cartilage areas. On the other end, in highly disrupted cartilage areas, large clusters were observed surrounded by small clusters and further away from the lesion by double strings. These large clusters were located mainly in the heart of cartilage injuries, often between extensive ECM fissures^16^. Interestingly, cluster formation never occurred in the superficial zone itself, but only in deeper tissue layers. This might be as the superficial zone is eroded during degeneration prior to such cluster formation.

An interesting observation in our study was the presence and location of double strings: These were present at the transition from intact cartilage to beginning cartilage degradation but they were also frequently found at the transition from the tide mark to the deep zone. This observation raises the hypothesis that there might be two types of double strings: one as previously described^10^ a result of cellular remodelling present in the adjacent tissue surrounding OA lesions, and one that arises from the base of the cartilage. It has already been described that the deep zone can act as a possible reservoir of chondroprogenitor cells^17^. These double strings arising from the base of the cartilage might thus be the structuro-morphological manifestation of regenerative cell proliferation and cartilage rebuilding.

Interestingly, in one sample, an additional spatial pattern was identified that seemingly acted as a plug or scar above an injured cartilage area where the chondrocytes lose higher spatial organisation but are found as densely disorganised diffuse cells. This diffuse cellular organisation has previously been proposed as end-stage OA with subsequent gradual cell loss^7,10^. The predominant pattern in regions of advanced tissue degeneration is that of large clusters. Although it is possible that they represent an effort of the cartilage to repair and maintain the remaining tissue^18^ it is generally believed that clusters contribute to OA progression and degradation of the joint^19,20^. In an inflammatory condition for example, too many cells in association with an altered phenotype might lead to excessive levels of inflammatory cytokines and catabolic enzymes, thus further disrupting the tissue or possibly leading to a self-perpetuating loop to promote further cell division^21^. This would explain the monstrous clusters containing several hundreds of cells that can sometimes be found. The extent or point in the cellular organisational pattern where the cartilage functionally still benefits from this increase in cells thus needs to be evaluated and will be the object of our future research.

Even though the exact reasons for the pattern changes have not yet been elucidated, several processes such as cell proliferation, apoptosis, matrix remodelling due to catabolic activity and pro-inflammatory effects, and directed cell migration^22–27^ have been suggested to be involved in this dynamic process. The distinct spatial patterns that the chondrocytes form and their distribution throughout articular cartilage may thus also reflect their functional interaction within a remodelling ECM. An interesting approach proposed by Kouri et al. in 1996 suggests that cells that are initially dispersed within the matrix can migrate towards one another and aggregate^28^. In fact, several in vitro studies showed that chondrocytes are capable of migrating under the stimulation of various chemoattractants^29–32^. It can, therefore, be speculated that OA-associated matrix degradation facilitates cellular migration allowing the rapid change of the cellular patterns.

Cellular density of the articular cartilage decreases sharply during growth and maturation of the skeleton, staying then relatively constant during adult life^33,34^. Interstingly, the OA associated changes accompanying cellular rearrangement appear to be accompanied by a change in local cellular density: We found high numbers of chondrocytes present in the superficial zone and the lowest cell numbers in microscopically intact deeper cartilage layers composed of single strings, which is consistent with the literature^33,35,36^. Of note, the more pathological the spatial pattern, the higher the cell count, which highlights the role of cell proliferation in the process of changing spatial chondrocyte patterns during OA.

With our three-dimensional approach we were thus able to support the physiopathological model suggested by Rolauffs for the superficial zone^6–8,10^ as a 3D-function of tissue degradation throughout all layers of articular cartilage. We also identified two different spatial presences of doubles strings raising the possibility that there might actually be two types of double strings—one as a result of cellular remodelling in the course of OA and one arising from the base of the cartilage as a possible structuro-morphological manifestation of regenerative proliferation and cartilage rebuilding.

### Study limitations

While SR-µCT offers a highly interesting and new perspective to OA analysis, the method is still hard to come by and with the presently available techniques it is very time consuming. For these reasons, only three osteoarthritic specimens were finally analysed. This should be borne in mind when interpreting the statistical results. Our findings are, however, highly consistent with the previous publications that analysed the presence, distribution and functional relevance of cellular rearrangement in osteoarthritic cartilage^6–10,12,13^. Dehydrating the cartilage samples may have distorted the morphology of the specimens. For this reason, we meticulously performed our immunohistochemical analyses on the same samples that were subjected to SR-µCT. We were thus able to match the wet fluorescence microscopy images with the dry SR-µCT-images to cross-validate that no major distortion had occurred during the dehydration. To allow a direct comparison, both techniques are presented in the same figure for each sample. Since cellular density analyses were performed on histological sections the numbers obtained are not volume-based and thus could be affected by different penetration depths of the microscope and overlapping of cells. Performing cell counting and cellular density assessment in the SR-µCT data would, however, also be prone to error in dehydrated samples due to tissue processing artefacts that in some cases lead to hyperdense areas where cell segmentation is not unambiguous. Phase-contrast SR-µCT imaging in entirely native samples might provide a solution to these yet unsolved obstacles.

## Conclusion

Performing 3D-analyses of spatial chondrocyte organisation by means of fluorescence microscopy and SR-µCT imaging, we were able to corroborate that changes in spatial chondrocyte organisation and its associated cellular density are hallmarks of OA-related tissue changes. The observed spatial changes appear to not just exist in the horizontal but also the vertical plane—thus having a 3D-function around cartilage lesions. They thus represent the “fingerprints” of the 3D-function of tissue destruction in OA.

## Materials and methods

### Cartilage samples

Tissue was obtained from patients who received total knee arthroplasty for symptomatic end-stage OA of the knee as radiologically judged by a Kellgren-Lawrence grade of 3 or 4^37^ in the Department of Orthopaedic Surgery of the University Hospital of Tübingen, Germany; in the BG Trauma Centre Tübingen, Germany; and in the Winghofer Clinic, Rottenburg, Germany. Additionally, one sample serving as healthy cartilage sample was analysed obtained from a 14-year-old patient receiving proximal tibial resection and joint replacement due to an osteosarcoma. Full departmental, institutional and local ethical committee approval were obtained before commencement of the study (project number of the ethics committee of the Medical Faculty/University of Tübingen 171/2014BO2). Written informed consent was received from all patients before participation. In case of the 14-year old patient, informed consent was obtained from both, the patient and the parents. All methods were performed in accordance with the relevant guidelines and regulations. This study was performed in accordance with the Declaration of Helsinki.

### Tissue preparation

Femoral condyle resections from the load-bearing area were selected according to their macroscopic degeneration pattern and washed in phosphate buffered saline (PBS) to remove remaining debris and blood cells. Cartilage-bone cuboids about 3.5 mm in width and 20 mm in length were cut from the condyles along the kinematic uniaxial movement direction of the knee. Cuboids were fixed in 4% paraformaldehyde/PBS (pH 7.0) for 24 h at 6 °C. Decalcification was performed in 20% (w/v) ethylenediaminetetraacetic acid/PBS at 37 °C for several days with solution changes every 2 days. Sufficient decalcification was determined by easy needle penetration of the bone. Final decision as to inclusion of the samples for the study including histologic and SR-µCT analysis was made using a Carl Zeiss Observer Z1 fluorescence microscope with the Axio Vision Release 4.8 software (Carl Zeiss Microscopy) after DAPI (Exmax 358 nm, Emmax 461 nm, Life Technologies) staining 0.1% (v/v)/PBS for 5 min. Tissue samples were considered to be suitable for the study if on a sample—within a distance of 2 cm—areas were contained that still had both intact cartilage surface as well as focal cartilage lesions.

### Section preparation and microscope image acquisition

Sectioning for side-view images of the cartilage was performed with a Leica cryotome, type CM3050S (Leica Biosystems, Wetzlar, Germany), at 70 µm thickness. For top-down view analyses, the remaining tissue block was processed as a whole. Mosaic fluorescent images were created with the AxioVision Release 4.8 software, including the MosaiX image acquisition software (Carl Zeiss Microscopy, Jena, Germany). Post-imaging processing of the images was performed with Adobe Illustrator and Adobe Photoshop (Adobe, San Jose, CA, USA). The side views of the cartilage were mapped in colour according to their locally predominant spatial pattern in order to visualise the connection between spatial organisation and tissue damage.

### *SR-µCT measurements *via* absorption contrast and image rendering post-tomography*

A detailed description of the imaging technique can be found in Supplementary File 1. In short, samples selected for SR-µCT were stained with propidium-iodide for better cellular contrast and then dehydrated. SR-µCT measurements were performed at the DESY (Deutsches Elektronen Synchrotron) IBL P05 imaging beamline Hamburg, Germany, operated by Helmholtz Zentrum Geesthacht. The final field of view was 3.7 × 3.7 mm^2^ with a beam height of 2 mm. The effective pixel size was 1.3 µm. Tomography was performed with 1200 angular steps at 180° rotation at 12 keV with an exposure time of 250 ms. Due to the limited beam height in combination with the length of the specimens, images were taken at subsequent height steps of 1.8 mm increments. The flat-field corrected scans were reconstructed using the filtered-back projection method applying a binning of two, resulting in a voxel size of 2.6 × 2.6 × 2.6 µm^3^. The final 8 bit format led to a total of 256 different possible grey values for each pixel. Data segmentation and 3D rendering was performed using Amira 6.0 (ThermoFisher Scientific, Waltham, MA, USA). Using the segmentation editor, the different spatial chondrocyte patterns were identified and selected. Selection was performed both manually by directly selecting the cells from their surrounding ECM and by means of applying a grey-value threshold, selecting the hyperdense cells (interval 39–255) and saving them into separate labels. The grey values from 0 to 38 were then defined as matrix-label. Artefact reduction and slight smoothing of the mesh surfaces were carried out using the 'smooth label' function in the segmentation editor of Amira and using unconstrained smoothing (kernel filter size set to 1 and minimal edge length set to zero). Surfaces were exported as Wavefront .obj file. Utilising the plugin 'Scientific3DFigurePDFApp'^38^ in the software package MeVisLab (MeVis Medical Solutions AG, Bremen, Germany) the corresponding .obj files were combined and colour coded and saved in U3D format. The latter was used to import the 3D-visualisations into a PDF file within the same Mevislab plugin. The CT-images of the cartilage were also mapped in colour according to their locally predominant spatial pattern in order to visualise the connection between spatial organisation and tissue damage.

### Statistical analysis of the histologic sections

To analyse cellular density per organisational pattern, three independent rectangles were plotted in each pattern-specific area in each of the four side-view sections for cell counting. These rectangles were distributed evenly in the targeted tissue areas available for each predominant spatial pattern. Due to the high heterogeneity of the cartilage surface and structure especially in areas with advanced tissue destruction, these rectangles could not be made of identical sizes. The rectangles were, however, only plotted once and then read out in a blinded fashion. Normality of the data was assessed by means of histograms. Because results were non-normally distributed, values are presented as median (interquartile range). Comparison between the different cell densities was performed by using the Kruskal–Wallis test. Post-hoc testing was performed with the Mann–Whitney U test. All reported p-values are two sided. A significance level of α = 0.05 was adjusted to the false discovery rate (FDR) by using the Benjamini–Hochberg procedure. Graphic illustration was performed by using boxplots. Statistical analysis was performed with SPSS Statistics 22 (IBM Corp., Armonk, NY, USA).

## Supplementary Information

Supplementary Information.

## Data Availability

Most data generated or analysed during this study are included in this published article (and its Supplementary Information files). The unedited raw data generated during and/or analysed during the current study are available from the corresponding author on reasonable request.

## Abbreviations

DS: Double strings
ECM: Extracellular matrix
Em: Emission
Ex: Excitation
LC: Large clusters
µCT: Micro-computed tomography
OA: Osteoarthritis
SC: Small clusters
SS: Single strings
SZ: Superficial zone
PBS: Phosphate buffered saline
FDR: False discovery rate
3D: Three-dimensional
