# Supplementary material for: Exploration of changes in spatial chondrocyte organisation in human osteoarthritic cartilage by means of 3D imaging
Source: Sci Rep. 2021 May 7;11:9783. doi: 10.1038/s41598-021-89582-w (PMC8105369; doi:10.1038/s41598-021-89582-w)
Supplement: Supplementary file 1 — Supplementary Information. [file 41598_2021_89582_MOESM1_ESM.pdf]

Title Page Document

Title: Exploration of changes in spatial chondrocyte organisation in human osteoarthritic cartilage by means of 3D imaging

Marina Danalache (PhD) (1)°	Email: danalachemarina@yahoo.com
Kevin Ralf Beutler (M.D.) (2,6)*°	Email: kevin.beutler@kbeutler.com<br>Phone: 0049 (0)7071-2986685
Bernd Rolauffs (M.D.) (3)	Email: berndrolauffs@googlemail.com
Julius Michael Wolfgart (2)	Email: julius-michael.wolfgart@student.uni-tuebingen.de
Florian Christof Bonnaire (M.D., B.Sc.) (1)	Email: florian.bonnaire@web.de
Stefan Fischer (PhD) (4,7)	Email: st.fischer@uni-tuebingen.de
Imke Greving (PhD) (5)	Email: imke.greving@hzg.de
Ulf Krister Hofmann (M.D.) (1)	Email: ulf.hofmann@med.uni-tuebingen.de

° Both authors contributed equally

* Corresponding author

(**1**) Department of Orthopaedic Surgery, University Hospital of Tübingen, Hoppe-Seyler-Strasse 3, D-72076 Tübingen, Germany

(**2**) Medical Faculty of the University of Tübingen, D-72076 Tübingen, Germany

(**3**) G.E.R.N. Tissue Replacement, Regeneration & Neogenesis, Department of Orthopedics and Trauma Surgery, Medical Center - Albert-Ludwigs-University of Freiburg, Faculty of Medicine, Albert-Ludwigs-University of Freiburg, D-79108 Freiburg, Germany

(**4**) Department of Evolutionary Biology of Invertebrates, University of Tübingen, D-72076 Tübingen, Germany; Present address: Tübingen Structural Microscopy (TSM), Center for Applied Geoscience (ZAG), University of Tübingen, D-72076 Tübingen, Germany

(**5**) Institute of Materials Research, Helmholtz-Zentrum Geesthacht, Geesthacht, Germany

(6) Present address: Department of Orthopaedic Surgery and Traumatology, Spital Thurgau AG, Spitalcampus 1, 8596 Münsterlingen, Switzerland

(7) Present address: Tübingen Structural Microscopy (TSM), Center for Applied Geoscience (ZAG), University of Tübingen, 72076 Tübingen, Germany.

Supplementary Figure legends

Supplementary Figure 1. Representative cellular organisational patterns from different areas of the degenerate cartilage. Similar to the arcade forming collagen fibres, single strings occur in a horizontal orientation in the superficial zone and ascend vertically in the deeper cartilage layers. Closer to the cartilage lesion, double strings can be observed. In close proximity to the defect, small clusters can be found that grow as they approach the heart of the lesion to form large clusters. In some areas near the focal cartilage defects, diffuse repair tissue can be found. This figure was created using Adobe Photoshop CS6 (https://www.adobe.com/ch_de/products/photoshop.html) and Adobe Illustrator CS6 (https://www.adobe.com/ch_de/products/illustrator.html).

Supplementary Figure 2. Segmented slices of cartilage of chondrocytes and extracellular matrix (A-D) and of the isolated chondrocytes (A'-D'). From an entire cartilage sample slices of interest were defined and isolated from the data file. In these slices a threshold-based labelling of extracellular matrix and chondrocytes was performed. The extracellular matrix is labelled in turquoise, the chondrocytes are labelled in pink. (**A/A'**) strings, (**B/B'**) double strings, (**C/C'**) small clusters, and (**D/D'**) large clusters. This figure was created using Amira 6.0 (https://www.thermofisher.com/ch/en/home/industrial/electron-microscopy/electron-microscopy-instruments-workflow-solutions/3d-visualization-analysis-software/amira-life-sciences-biomedical.html) and Adobe Illustrator CS6 (https://www.adobe.com/ch_de/products/illustrator.html).

Supplementary Figure 3. Identification of individual patterns in a cartilage data set. Sagittal renderings of cartilage specimen I (Figure 3) (A, B). Individual spatial chondrocyte patterns were selected and segmented for visualisation (C1-4). Their localisation in the cartilage sample is indicated by the coloured lines and squares. Chondrocytes are labelled in pink, their lacunae in dark blue. Scaling adjusted in C1-C4 to maximise visibility of each pattern. This figure was created using Amira 6.0 (https://www.thermofisher.com/ch/en/home/industrial/electron-microscopy/electron-microscopy-instruments-workflow-solutions/3d-visualization-analysis-software/amira-life-sciences-biomedical.html) and Adobe Illustrator CS6 (https://www.adobe.com/ch_de/products/illustrator.html).

Supplementary Figure 4. Sagittal (A) and axial (B) renderings of the cartilage sample I from figure 4. The orange line in (A) is the scout indicating the position of (B). In the left part of the sagittal image the cartilage is still intact throughout all layers. The nuclei can be identified in their hypodense lacuna of cytoplasm. In the right part of the sagittal image the cartilage is already strongly compromised with a reduction in height, and formation of fissures and large clusters. The retiform grid below the cartilage is the subchondral bone. This figure was created using Amira 6.0 (https://www.thermofisher.com/ch/en/home/industrial/electron-microscopy/electron-microscopy-instruments-workflow-solutions/3d-visualization-analysis-software/amira-life-sciences-biomedical.html) and Adobe Illustrator CS6 (https://www.adobe.com/ch_de/products/illustrator.html).

Supplementary Figure 5. Pattern specific chondrocyte density. Cartilage specimens from Figures 1 (**A**), 3 (**B**), 4 (**C**), and 5 (**D**) with labelled pattern-specific rectangles for cell counting for comparison of cellular density as a function of cellular organisation. This figure was created using Adobe Photoshop CS6 (https://www.adobe.com/ch_de/products/photoshop.html) and Adobe Illustrator CS6 (https://www.adobe.com/ch_de/products/illustrator.html).

Supplementary Tables

Supplementary Table 1. Comparison of cellular density as a function of cellular organisation by using the false-discovery rate (FDR) for alpha adjustment

Comparison	p-value	FDR-adjusted alpha
All groups	**0.000001**	**0.000220**
SZH-SSH	**0.000041**	**0.000751**
SZH-SZ	**0.000041**	**0.000751**
SZH-SS	**0.000041**	**0.000751**
SZH-DS	**0.000041**	**0.000751**
SZH-SC	**0.000082**	**0.000128**
SZH-LC	**0.010613**	**0.013734**
SSH-SZ	0.258083	0.258083
SSH-SS	**0.000041**	**0.000751**
SSH-DS	**0.000082**	**0.000128**
SSH-SC	0.258083	0.258083
SSH-LC	**0.003990**	**0.005486**
SZ-SS	**0.000041**	**0.000751**
SZ-DS	**0.000041**	**0.000751**
SZ-SC	0.222419	0.244660
SZ-LC	**0.024434**	**0.029863**
SS-DS	0.062526	0.072398
SS-SC	**0.000041**	**0.000751**
SS-LC	**0.000041**	**0.000751**
DS-SC	**0.000041**	**0.000751**
DS-LC	**0.000041**	**0.000751**
SC-LC	**0.000165**	**0.000242**

For graphic display, see also Figure 6. Abbreviations: SZH - superficial zone healthy cartilage; SSH - single strings healthy cartilage; SZ - superficial zone; SS - single strings; DS - double strings; SC - small clusters; LC - large clusters. Significant p-values are denoted in bold.

Supplementary Figures

Supplementary Figure 1

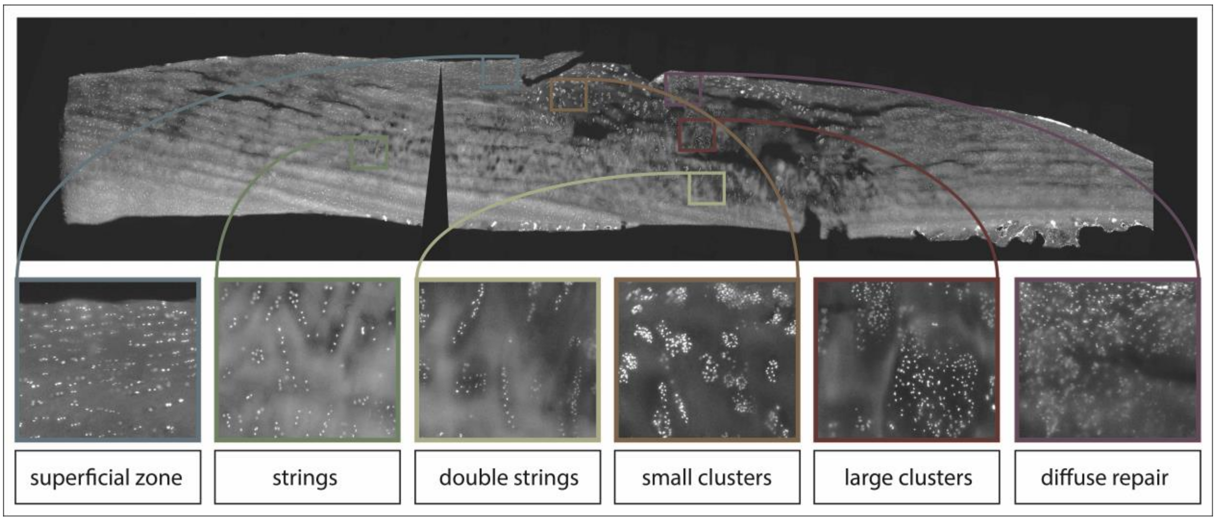

92 **Supplementary Figure 2**

93 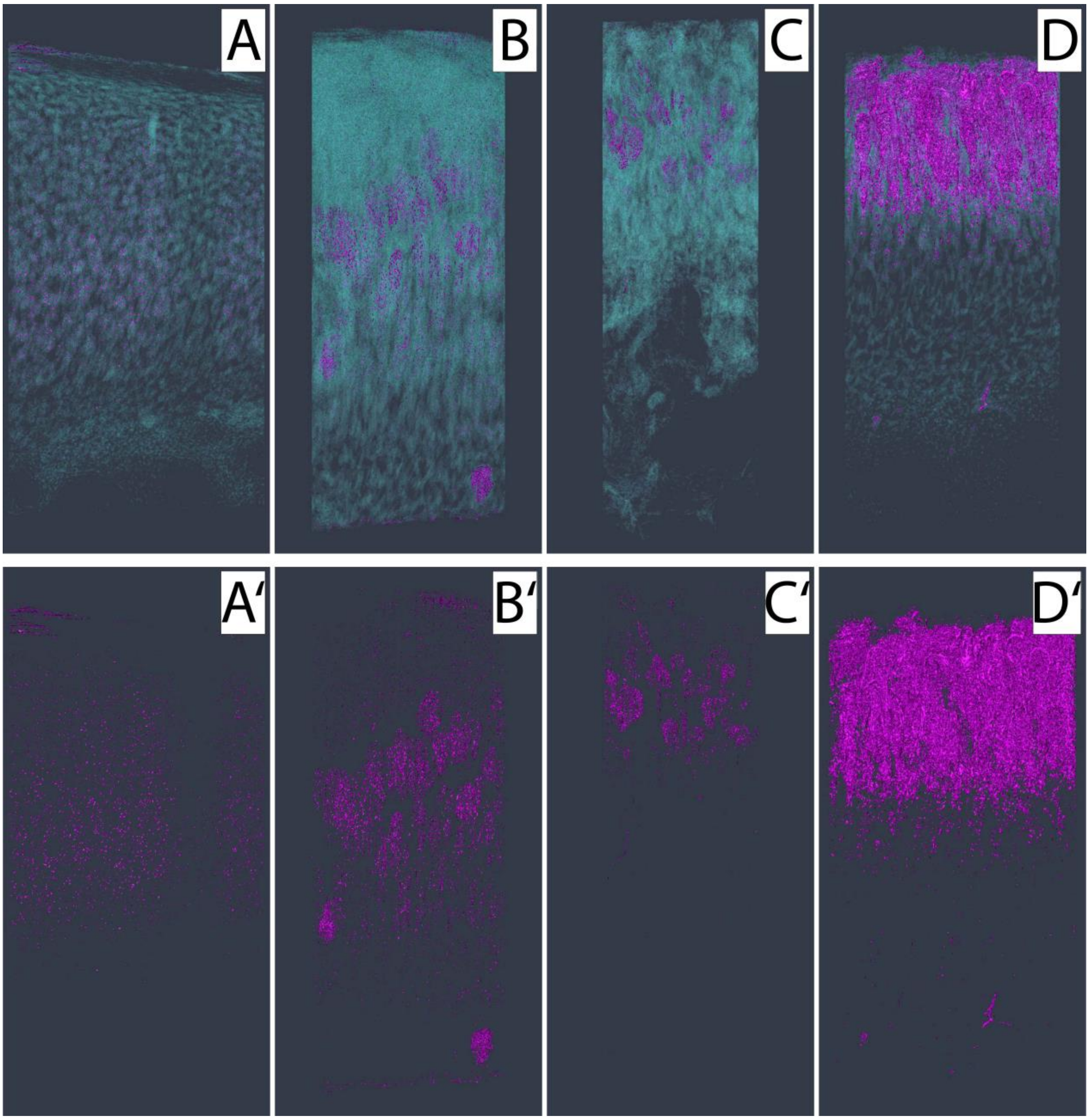

94

Supplementary Figure 3

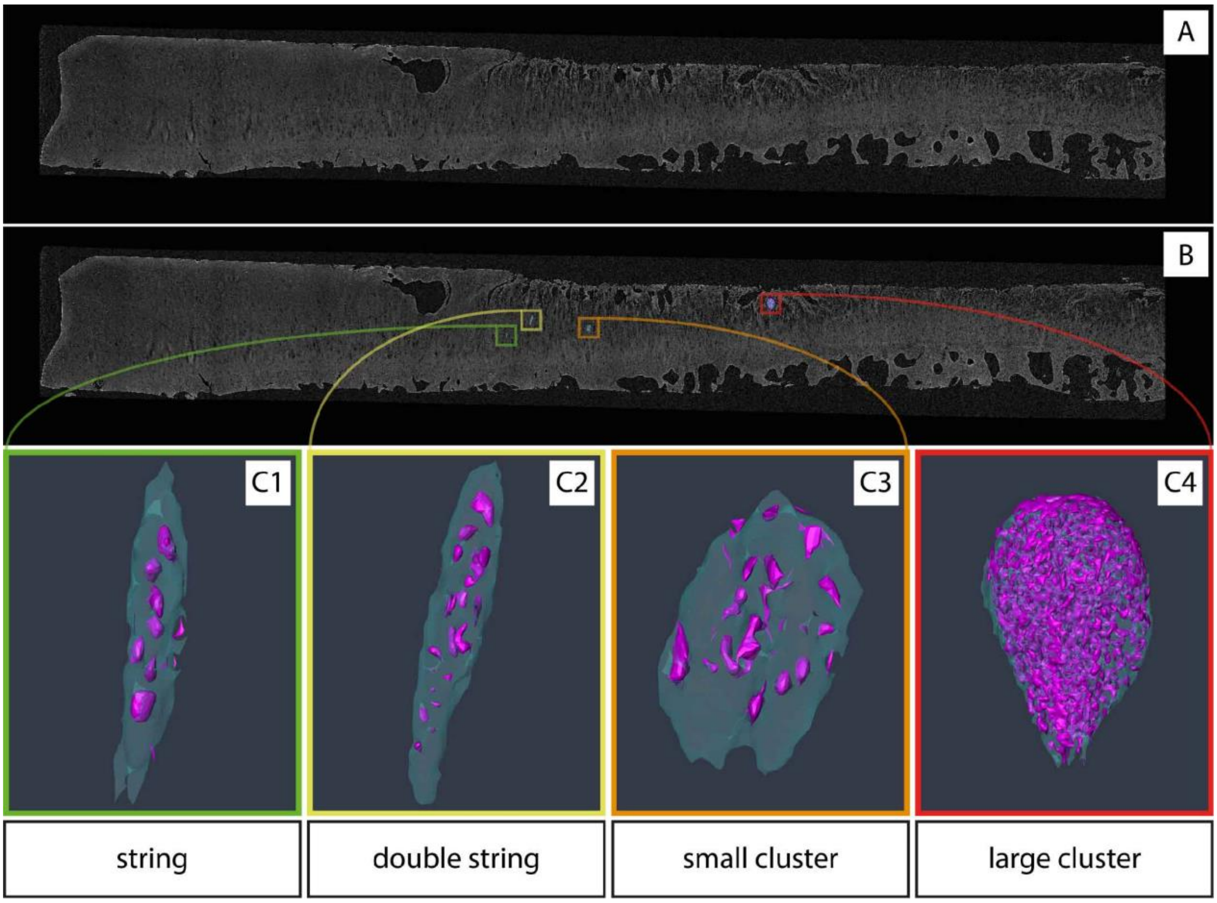

Supplementary Figure 4

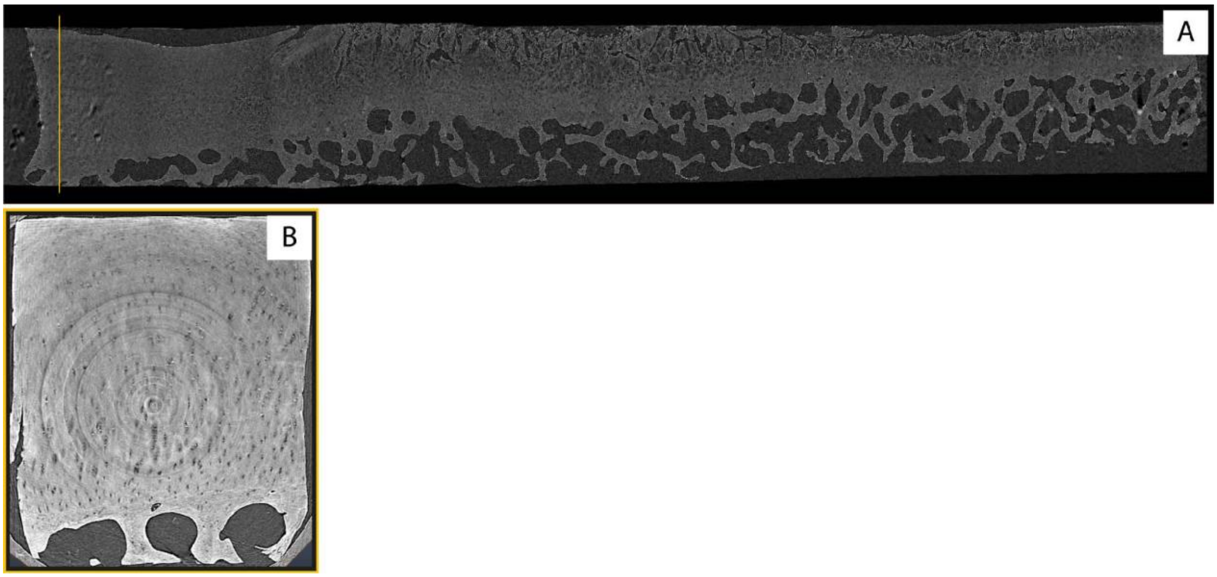

101 **Supplementary Figure 5**

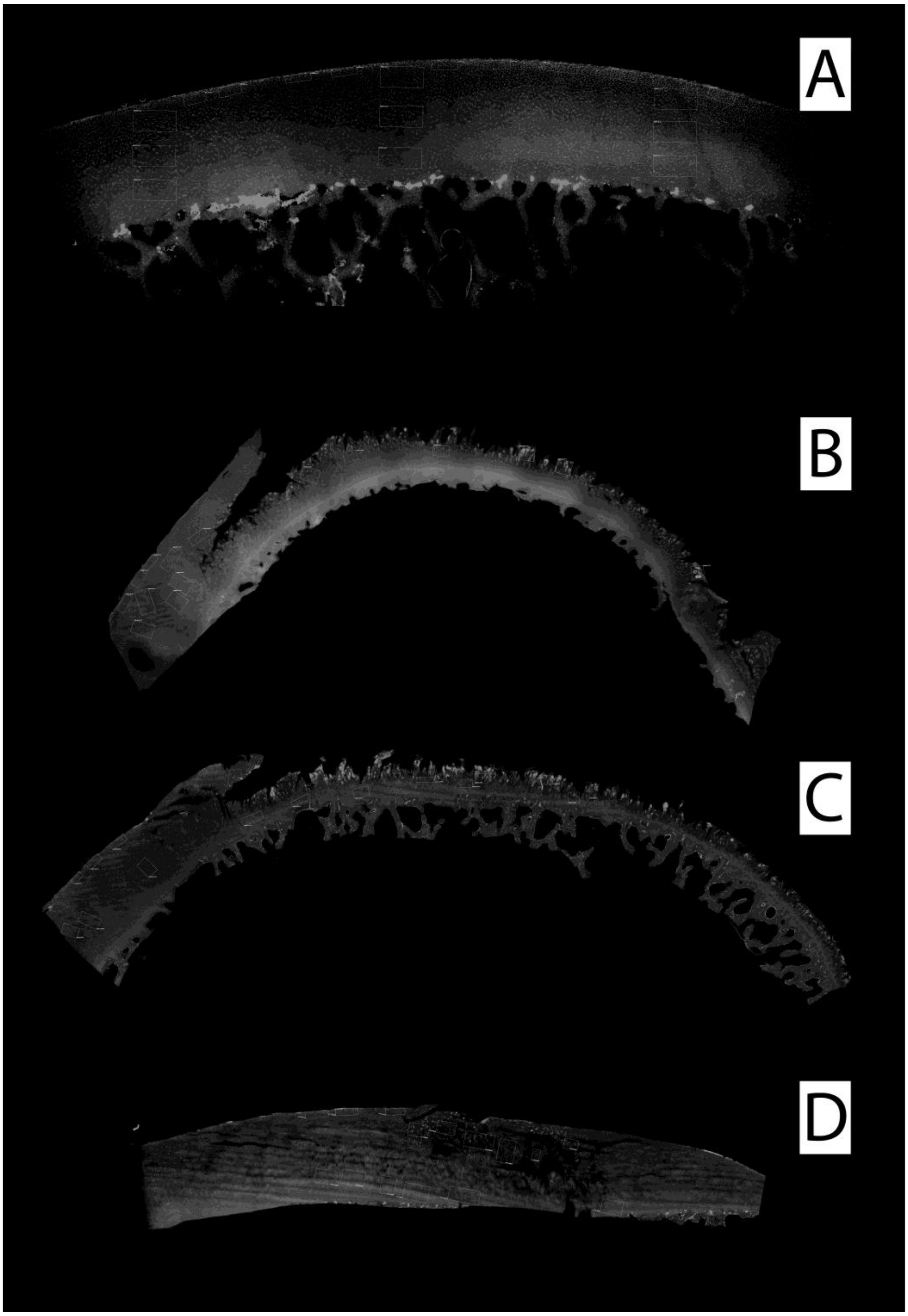

102

Supplementary Methods

SR-μCT measurements via absorption contrast

Several recent publications have been analysing the musculoskeletal system in various animal models (i.e.: mouse [1-3], rat [4,5], rabbit [6], goat [7], and horse [8,9]) by means of SR-μCT. Those studies that used human tissue for their analyses were to date still limited to a supracellular resolution[10-12]. Zehbe et al. and Clark et al. had, however, already demonstrated the feasibility of identifying single chondrocytes using synchrotron radiation micro-computed tomography (SR-μCT) in bovine intact cartilage with a spatial resolution of approximately 2 μm [13-15]. To the knowledge of the authors, no study has yet analysed the 3D chondrodyte organisation in human degenerative cartilage.

For better cellular contrast, samples selected for SR-μCT were stained with propidium-iodide (Roche-Diagnostics) 1 μl/0.2 ml PBS for 24 hours at 6°C. After 3x30 min PBS washes on the nutator, samples were dehydrated in ethanol/PBS with an increase in ethanol concentration every 6 hours (25, 50, 75, 90, 95, 100%). Specimens were finally treated twice with Xylene (30 min), whereafter samples were left under the hood for drying. Tissue samples were stored in dry atmosphere in a standard eppendorf tube using silica gel (Wisemini, Wisepac Europe GmbH, Bochum, Germany) as a desiccant.
SR-μCT measurements were performed at the DESY (Deutsches Elektronen Synchrotron) IBL P05 imaging beamline Hamburg, Germany, operated by Helmholtz Zentrum Geesthacht. Detailed specifications of the instrument are described elsewhere [16]. The beamline is equipped with two different monochromators - one double crystal monochromator (DCM) and one double multilayer monochromator (DMM). For this experiment the DCM was chosen, allowing for an energy range of 5-50 keV with an energy spread of 10^{-4}. The experimental setup was thus optimised for attenuation-based imaging and allows for high spatial and density resolution. The 100 μm thick CdWO4 crystal was mounted in front of the optics of

the camera tower. The visible light was guided over a mirror system into a CCD camera (EHD SC-9000/Kodak KAF 09000 chip) with 12x12 μm^2 pixel size and the chosen magnification was 10x, resulting in a field of view of 3.7x3.7 mm^2 with a beam height of 2 mm. The effective pixel size was 1.3 µm, sample-detector distance was 2 cm and a decoherer was installed to minimise phase effects. Tomography was performed with 1200 angular steps at 180° rotation. Best absorption contrast was found to be at 12 keV with an exposure time of 250 ms.

Before measurements, samples were carefully transferred into standard plastic straws, vertically aligned and glued onto the specific metal sample holders. These were then left in the air-condition temperature regulated experimental hutch for acclimatisation for at least 1 hour. Thereafter, samples were placed into the high-precision air-bearing rotation stage for scanning. Due to the limited beam height in combination with the length of the specimens, images were taken at subsequent height steps of 1.8 mm increments. This allowed sufficient overlap of the stacked data of 100 µm.

The flat-field corrected scans were reconstructed using filtered-back projection method applying a binning of two, resulting in a voxel size of 2.6x2.6x2.6 μm^3. The initially generated single 'spectral library images' were imported into the open source software 'ImageJ' (The National Institutes of Health, US) and transformed into 'tagged images files' (TIFF).

Image rendering post-tomography

The original TIFF 32 bit image stack, generating a file size of about 85 GB was reduced to 8 bit format before segmentation, in order to handle the complete project file on the reconstruction computer. This lead to a total of 256 different possible grey values for each pixel. The data set was furthermore cropped to the image volume containing the actual cartilage piece in Fiji [17], by that obtaining a final file size of about 20 GB. Data segmentation

and 3D rendering was performed using Amira 6.0 (ThermoFisher Scientific, Waltham, MA, USA). Using the segmentation editor, the different spatial chondrocyte patterns were identified and selected. Selection was performed both manually by directly selecting the cells from their surrounding ECM and by means of applying a grey-value threshold, selecting the hyperdense cells (interval 39-255) and saving them into separate labels. The grey values from 0-38 were then defined as matrix-label. The combination of manual and automated selection is necessary, as there is no single threshold. The combination of value settings for surface generation and smoothing were tested in advance and compared to meshes generated on raw unprocessed labels (data not shown). A certain amount of smoothing of the surfaces is needed in order to keep the number of vertices in the meshes in a certain range to keep the the size of the meshes manageable within the software. The mesh sizes (number of points and faces of the vertices) of extremely rough surfaces (by single noise pixels etc.) are multiple times higher, which easily goes into the limits of the processability of these meshes with high performance imaging workstations.

Artefact reduction and slight smoothing of the mesh surfaces were carried out in a two fold way, first using the 'smooth label' function in the segmentation editor of Amira (removes noise (few pixel) on the label outline of the object in each image plane) and secondly using unconstrained smoothing (kernel filter size set to 1 and minimal edge length set to zero (no decrease in the number of mesh triangles)) during surface generation (algorithm by Hege et al. [18]) and surface smoothing (algorithm by Taubin [19]; using 60 iterations and a lambda value of 0.3) during the subsequent generation of the 3D mesh surfaces. All labels and surfaces were visually checked against the original image data using the Quad-View in Amira, to ensure that no excessive smoothing or loss of volume occurred. In a final step in Amira, surfaces were exported as Wavefront .obj file. Utilising the plugin 'Scientific3DFigurePDFApp' [20] in the software package Mevislab (MeVis Medical Solutions

AG, Bremen, Germany) the corresponding .obj files were combined and colour coded and saved in U3D format. The latter was used to import the 3D-visualisations into a PDF file within the same Mevislab plugin. The CT-images of the cartilage were also mapped in colour according to their locally predominant spatial pattern in order to visualise the connection between spatial organisation and tissue damage.

References

1 Zheng, H. *et al.* 802-812 (Springer International Publishing).

2 Sun, J. *et al.* Pyk2 deficiency enhances bone mass during midpalatal suture expansion. *Orthod Craniofac Res* **23**, 501-508, doi:10.1111/ocr.12402 (2020).

3 Gabner, S., Böck, P., Fink, D., Glösmann, M. & Handschuh, S. The visible skeleton 2.0: phenotyping of cartilage and bone in fixed vertebrate embryos and foetuses based on X-ray microCT. *Development* **147**, dev187633, doi:10.1242/dev.187633 (2020).

4 Milovanovic, P. *et al.* Moderate hyperhomocysteinemia induced by short-term dietary methionine overload alters bone microarchitecture and collagen features during growth. *Life Sci* **191**, 9-16, doi:10.1016/j.lfs.2017.10.008 (2017).

5 Donato, S. *et al.* Meniscal Ossicles as micro-CT Imaging Biomarker in a Rodent Model of Antigen-Induced Arthritis: a Synchrotron-Based X-ray Pilot Study. *Scientific Reports* **7**, 7544, doi:10.1038/s41598-017-08025-7 (2017).

6 Fernández-Martín, S., Permuy, M., López-Peña, M., Muñoz, F. & González-Cantalapiedra, A. No Effect of Long-Term Risedronate Use on Cartilage and Subchondral Bone in an Experimental Rabbit Model of Osteoarthritis. *Front Vet Sci* **7**, 576212, doi:10.3389/fvets.2020.576212 (2020).

7 Blom, R. P., Mol, D., van Ruijven, L. J., Kerkhoffs, G. M. M. J. & Smit, T. H. A Single Axial Impact Load Causes Articular Damage That Is Not Visible with Micro-Computed Tomography: An Ex Vivo Study on Caprine Tibiotalar Joints. *CARTILAGE* **0**, 1947603519876353, doi:10.1177/1947603519876353 (2019).

8 Malekipour, F., Oetomo, D. & Lee, P. V. Subchondral bone microarchitecture and failure mechanism under compression: A finite element study. *J Biomech* **55**, 85-91, doi:10.1016/j.jbiomech.2017.02.005 (2017).

9 Malekipour, F., Whitton, C. R. & Lee, P. V. Stiffness and energy dissipation across the superficial and deeper third metacarpal subchondral bone in Thoroughbred racehorses under high-rate compression. *J Mech Behav Biomed Mater* **85**, 51-56, doi:10.1016/j.jmbbm.2018.05.031 (2018).

10 Delecourt, C. *et al.* Cartilage morphology assessed by high resolution micro-computed tomography in non OA knees. *Osteoarthritis and cartilage* **24**, 567-571, doi:10.1016/j.joca.2015.10.009 (2016).

11 Reina, N. *et al.* BMI-related microstructural changes in the tibial subchondral trabecular bone of patients with knee osteoarthritis. *J Orthop Res* **35**, 1653-1660, doi:10.1002/jor.23459 (2017).

12 Honkanen, M. K. M. *et al.* Synchrotron MicroCT Reveals the Potential of the Dual Contrast Technique for Quantitative Assessment of Human Articular Cartilage Composition. *J Orthop Res* **38**, 563-573, doi:10.1002/jor.24479 (2020).

13 Zehbe, R. *et al.* Going beyond histology. Synchrotron micro-computed tomography as a methodology for biological tissue characterization: from tissue morphology to

individual cells. *Journal of the Royal Society, Interface / the Royal Society* **7**, 49-59, doi:10.1098/rsif.2008.0539 (2010).
14 Zehbe, R., Riesemeier, H., Kirkpatrick, C. J. & Brochhausen, C. Imaging of articular cartilage--data matching using X-ray tomography, SEM, FIB slicing and conventional histology. *Micron* **43**, 1060-1067, doi:10.1016/j.micron.2012.05.001 (2012).
15 Clark, J. N. *et al.* Propagation phase-contrast micro-computed tomography allows laboratory-based three-dimensional imaging of articular cartilage down to the cellular level. *Osteoarthritis and cartilage* **28**, 102-111, doi:https://doi.org/10.1016/j.joca.2019.10.007 (2020).
16 Greving, I. *et al. P05 imaging beamline at petra III - First results*. Vol. 9212 (2014).
17 Schindelin, J. *et al.* Fiji: an open-source platform for biological-image analysis. *Nat Methods* **9**, 676-682, doi:10.1038/nmeth.2019 (2012).
18 Hege, H., Stalling, D., Seebafl, M. & Zˆckler, M.
19 Taubin, G. in *Proceedings of IEEE International Conference on Computer Vision.* 852-857.
20 Newe, A. Enriching scientific publications with interactive 3D PDF: an integrated toolbox for creating ready-to-publish figures. *PeerJ Comput. Sci.* **2**, e64 (2016).
